# Dynamic Resource Partitioning in a Marine Predator Community

**DOI:** 10.1002/ece3.74579

**Published:** 2026-11-02

**Authors:** T. Carpenter‐Kling, M. Connan, R. R. Reisinger, A. B. Makhado, P. G. Ryan, P. A. Pistorius

**Affiliations:** ^1^ Marine Apex Predator Research Unit (MAPRU), Department of Zoology, Institute for Coastal and Marine Research Nelson Mandela University Gqeberha South Africa; ^2^ DST‐NRF Centre of Excellence at the FitzPatrick Institute of African Ornithology Nelson Mandela University Gqeberha South Africa; ^3^ School of Ocean and Earth Science University of Southampton, National Oceanography Centre Southampton Southampton UK; ^4^ Oceans and Coasts Research, Department of Forestry, Fisheries and the Environment Cape Town South Africa; ^5^ FitzPatrick Institute of African Ornithology University of Cape Town Rondebosch South Africa

**Keywords:** fur seals, niche partitioning, seabirds, stable isotopes, sub‐Antarctic, trophic dynamics

## Abstract

To avoid competition, species partition resource‐use along various niche axes. However, variable resource availability, especially within dynamic environments, may influence this partitioning. Theory predicts that species will contract their ecological niche to limit competition during times of resource scarcity. We analysed blood δ^13^C and δ^15^N composition in four albatrosses, four penguin, two giant petrel and two fur seal species breeding on sub‐Antarctic Marion Island, during two seasons (autumn and summer) over 3 years. Using the species' seasonal stable isotopic niche space as a proxy for trophic niche space, we investigated their trophic dynamics and maintenance of inter‐specific resource partitioning. There were significant variations in the seasonal and inter‐annual trophic niches of these predators. However, despite this high seasonal and annual variability, resource partitioning among the species was maintained over time. Between years, there was greater variability in the species' trophic niches in autumn than in summer. This might reflect species being better able to track productive dynamic oceanographic features during autumn, when most species are less spatially constrained by breeding commitments compared to during summer. Our results indicate that most of these marine predators have foraging behaviours that are either sufficiently flexible or general to cope with resources that are variable in space and time. Their foraging strategies minimise inter‐specific overlap in food or foraging habitat preferences, potentially resulting from historic or present competition for resources.

## Graphical abstract

This manuscript uses blood carbon (δ^13^C) and nitrogen (δ^15^N) isotopic composition collected from an impressive 12 marine predators (sub‐Antarctic seabirds and seals) during two seasons over 3 years to test central theories in foraging ecology. By using the species' isotopic niche space as a proxy for trophic niche space, we investigate the consistency (i.e., interannual) trophic niche dynamics and partitioning of a predator community during two seasons (autumn and summer) of different life‐history stages and consequently, foraging constraints. This manuscript provides evidence for theories such as competitive exclusion principle and classical niche theory, both of which predict that in order to coexist species must partition their niches.

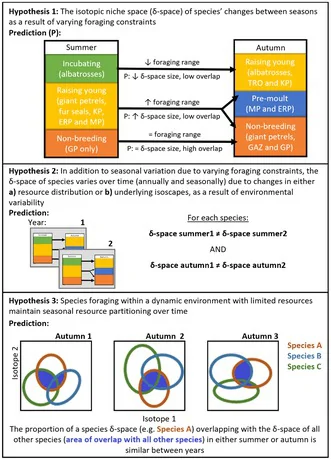

## 1 Introduction

Resource segregation among species is typically viewed in light of the competitive exclusion principle, which posits that competitors for identical resources cannot co‐exist (Gause 1934; Hardin 1960). Theoretically, species should limit overlap along various resource‐use, temporal and spatial axes. In dynamic environments, however, species also respond to fluctuations in resource availability to maintain fitness (Niella et al. 2025; Riotte‐Lambert and Matthiopoulos 2019; van Moorter et al. 2013). According to optimal foraging theory, when food resources are abundant, animals will specialise their foraging behaviour and target preferred food items and habitats, while during food scarcity they will generalise their foraging behaviour by consuming a greater diversity of food items (Perry and Pianka 1997; Stephens and Krebs 1986). However, when resources are limited, competition among ecologically similar species is thought to increase, promoting a decrease in interspecific niche overlap (Pianka 1974). Thus, classical niche theory (Hutchinson 1957; Pianka 1974; Schoener 1974) predicts that during periods of relative food scarcity, animals will adjust their foraging behaviour to decrease interspecific competition for resources by becoming more specialised. However, how these predictions operate in temporally variable environments remains poorly resolved (Ekrem et al. 2025; Manlick et al. 2019).

When considering the rapid rate of environmental changes globally, it is hardly surprising that many ecological studies are underpinned by a temporal dimension. However, niche theory is still most often applied as a static framework, despite the inherently dynamic nature of ecological systems. As such, changes in niche overlap and resource partitioning over time within sympatric animal communities remain poorly understood. This is problematic because animals often face intra‐ and inter‐annual variability in habitat and prey availability through environmental fluctuations and life‐history‐dependent foraging constraints (Bourgeois et al. 2022; Dehnhard et al. 2016; Phillips et al. 2017; Sydeman et al. 2015). Understanding the coexistence of multispecies assemblages, particularly those that live in seasonal environments, therefore requires explicit consideration of temporal variation in community‐level interactions.

To address this shortfall, we tested three hypotheses: (1) the size of species' stable isotopic niche space (δ‐space) varies seasonally in response to changing foraging constraints; (2) the position of species' δ‐space shifts seasonally and inter‐annually due to temporal changes in resource distribution and/or underlying stable isotopic baselines; and (3) despite temporal variability in species‐specific stable isotopic niches, community‐level resource partitioning is maintained over time in a resource‐limited system.

To test these three hypotheses, focusing on dynamic resource use at a community level, we studied land‐breeding marine predators, seabirds and fur seals, on a sub‐Antarctic island. During summer, multispecies assemblages, often numbering in the millions, aggregate on sub‐Antarctic islands to breed (e.g., Ryan and Bester 2008). The coexistence of these species has largely been attributed to partitioning of their trophic niches (Cherel et al. 2007; Connan et al. 2014; Jaeger et al. 2010, 2013; Pickett et al. 2018), with a greater degree of partitioning evident within seabird taxonomic groups (Connan et al. 2019; see Appendix S1 for simplified graphic illustration of dietary overlap). The ability of marine predators to reach distant foraging habitats varies during the breeding season, because time available for foraging at sea is primarily determined by the fasting capabilities of their partners or offspring. Furthermore, due to the aggregation of land‐breeding predators foraging from a central location, prey availability is thought to decline outward from these central locations as the breeding season progresses, likely increasing local competition for prey (Ashmole 1963; Furness and Birkhead 1984). As the breeding season progresses (and compared to the non‐breeding period), seabird species contract and further segregate their trophic niches in response to potential increased resource competition and lower prey availability (Barger et al. 2016; Cherel and Hobson 2007; Cherel et al. 2008), consistent with the prediction of classical niche theory. However, prey distributions also vary inter‐annually due to the dynamic nature of the marine environment (Lévy et al. 2015; Scales et al. 2014). Marine predators can buffer themselves against prey limitation by prey switching if they are generalists (Carpenter‐Kling, Reisinger, et al. 2020; Dehnhard et al. 2016) or alternatively through adopting flexible foraging behaviours (Carpenter‐Kling, Handley, et al. 2019; Carpenter‐Kling, Reisinger, et al. 2020; Mills et al. 2020; Phillips et al. 2019). However, some species show limited plasticity and track preferred habitats over time (e.g., major frontal zones; Bost et al. 2015).

Here, we investigate the trophic niche dynamics and maintenance of inter‐specific resource partitioning among 12 marine predator species breeding in sympatry on sub‐Antarctic Marion Island in the southern Indian Ocean. During autumn and summer over three consecutive years (2015/2016–2017/2018), we measured the carbon (^13^C/^12^C; δ^13^C) and nitrogen (^15^N/^14^N; δ^15^N) stable isotopic composition of the predators' whole blood to estimate their stable isotopic niche as a proxy for their trophic niche, with δ^13^C broadly indicating spatial distribution and δ^15^N indicating trophic position within an ecosystem (Newsome et al. 2007; Petalas et al. 2024). Because flying and diving predators are inherently segregated by foraging mode (diving vs. flying) and additionally flying seabirds are known to scavenge more often than actively hunt live prey, whereas diving species actively hunt live prey and very rarely scavenge (Banda 2019; Brown et al. 1990; Carpenter‐Kling, Handley, et al. 2019; Connan et al. 2014; Cooper et al. 1992; Hunter and Brooke 1992; Reisinger et al. 2018), we assessed the maintenance of resource partitioning within these two functional groups. The flying species included four albatrosses (wandering *Diomedea exulans* , grey‐headed *Thalassarche chrysostoma* , sooty *Phoebetria fusca* and light‐mantled *P. palpebrata* albatrosses) and two giant petrels (northern *Macronectes halli* and southern *M. giganteus* giant petrels), while diving species included four penguins (king *Aptenodytes patagonicus* , gentoo *Pygoscelis papua* , macaroni *Eudyptes chrysolophus* and eastern rockhopper *E. filholi* penguins) and two fur seals (sub‐Antarctic *Arctocephalus tropicalis* and Antarctic *A. gazella* fur seals).

Within the above context, and following from our three hypotheses, we predicted that (1) the stable isotopic niche size varies seasonally (summer versus autumn) as a result of varying foraging constraints (Figure 1, Hypothesis 1), and (2) the position and size of the stable isotopic niches vary seasonally and/or inter‐annually due to temporal changes in resource distribution (Figure 1, Hypothesis 2a), and/or variation in the underlying spatial isoscapes (Figure 1, Hypothesis 2b; Magozzi et al. 2017; St John Glew et al. 2021). The latter prediction was largely motivated by pronounced environmental variability over our study period including large latitudinal movement (~1°–2°) of major oceanographic fronts during 2015 (Carpenter‐Kling, Reisinger, et al. 2020). Lastly, we predicted that (3) despite temporal variation in the position of stable isotopic niches, the proportion of overlap among species' stable isotopic niches remains consistent through time, indicating persistent community‐level resource partitioning. Such stability would reflect evolutionary and behavioural adaptations that facilitate coexistence in a dynamic environment (Carpenter‐Kling, Reisinger, et al. 2020; Connan et al. 2014; Pickett et al. 2018; Wakefield et al. 2009).

**FIGURE 1:**
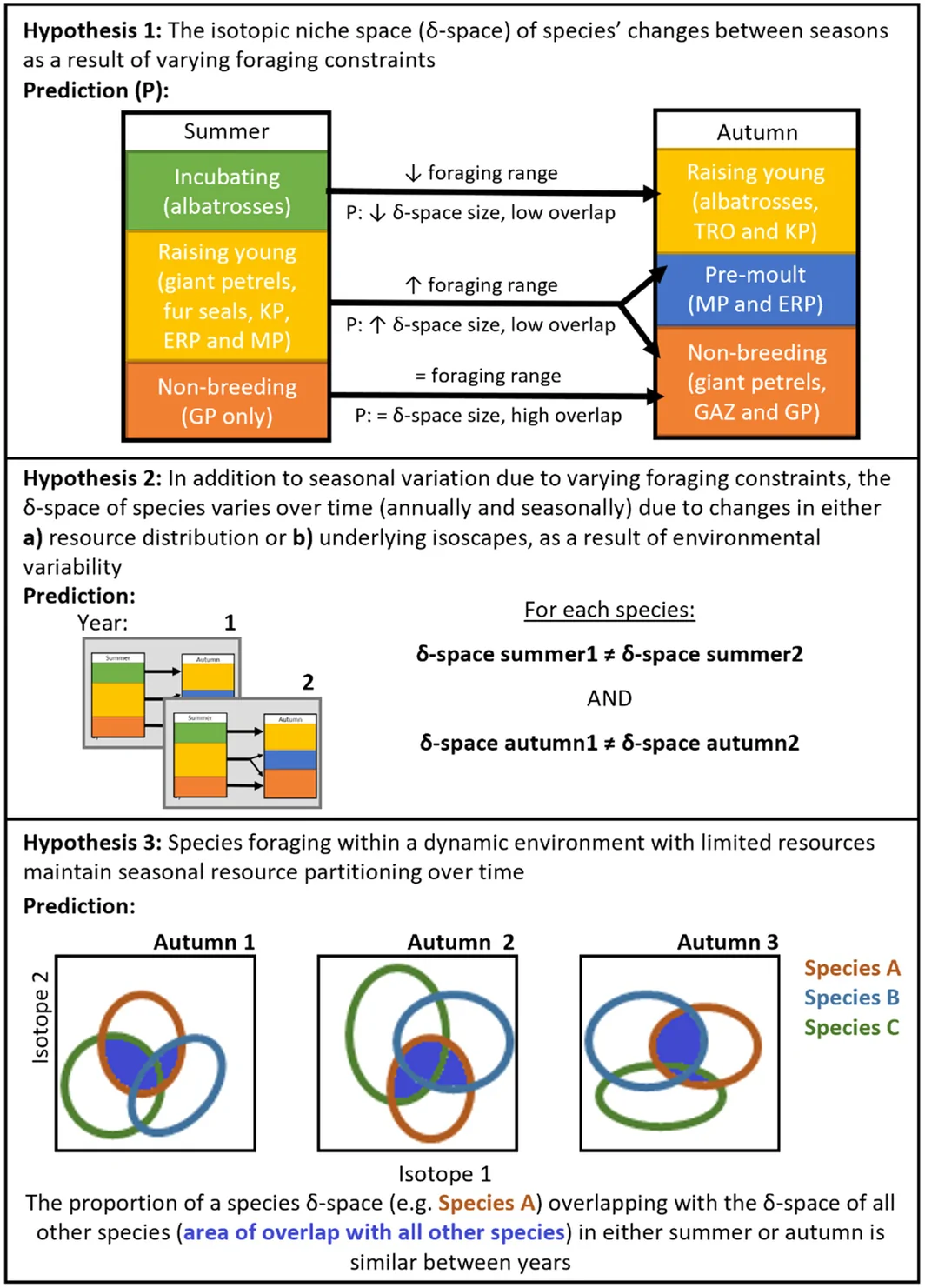
Schematic diagram of the three main hypotheses detailing seasonal and inter‐annual temporal variability in the stable isotopic niche space of four albatross (wandering, grey‐headed, sooty and light‐mantled albatrosses), two giant petrel (northern and southern giant petrels), four penguin (king KP, gentoo GP, macaroni MP and eastern rockhopper ERP penguins) and two fur seal species (Antarctic GAZ and sub‐Antarctic TRO fur seals).

## 2 Materials and Methods

### 2.1 Study Species and Sample Collection

Over three consecutive years, up to 20 individuals of each species were sampled along the south and south‐east coast of Marion Island (46.94° S, 37.86° E) during two seasons: autumn (April 2015, 2016 and 2017) and the following summer (December–February 2015/2016, 2016/2017 and 2017/2018) with a few exceptions due to differing annual breeding chronology of wandering albatross and giant petrels and low numbers of light‐mantled albatross adults present during autumn (Appendix S2). As a result, not all species were sampled in all seasons or years due to fieldwork constraints.

Approximately 0.5 mL of blood was collected per individual using a heparinized needle and immediately stored in 70% ethanol and frozen until preparation for stable isotope analysis (Hobson et al. 1997). For details of stable isotope analysis, refer to Appendix S3. Whole blood has a rapid turnover rate and represents an animal's diet integrated over a period of 2–4 weeks prior to sampling in seabirds and potentially longer in mammals (Dalerum and Angerbjörn 2005; Hilderbrand et al. 1996; Hobson and Clark 1992). Thus, the samples collected during summer give dietary information for incubating albatrosses, giant petrels and eastern rockhopper penguins, brooding macaroni penguins, lactating fur seals and non‐breeding gentoo penguins (Appendix S2). The samples collected during autumn give dietary information of early chick rearing wandering albatross, mid‐late chick rearing grey‐headed and sooty albatrosses, king penguin provisioning large chicks, non‐breeding giant petrels, Antarctic fur seals and gentoo penguins, pre‐moult foraging trip of the eastern rockhopper and macaroni penguins and lactating sub‐Antarctic fur seals (Appendix S2).

### 2.2 Statistical Analyses

Analyses were conducted in R (R Core Team 2024). To visualise species' stable isotopic niches (i.e., δ‐space) through time, standard ellipse areas from the δ^13^C and δ^15^N blood values with a 95% confidence interval (R package *SIBER*; Jackson et al. 2011) were estimated and plotted for each season and year (further referred to as sampling period). Intrinsic factors such as sex and age might influence the foraging ecology of species (e.g., Nel et al. 2001; Orgeret et al. 2020; Reisinger et al. 2020; Votier et al. 2017), however, due to insufficient sample sizes, such factors were not included as covariates in the analyses.

Hypothesis 1 *The δ‐space of animals is variable between seasons*.

Hypothesis 2a *The δ‐space of animals is variable among years*.

To investigate the expansion or contraction of each species' δ‐space, the area of the δ‐space during each sampling period was estimated and compared within species using Bayesian standard ellipse areas (SEAb). SEAb provide robust estimates of niche width that account for differences in sample size (Jackson et al. 2011). The probability of SEAb being larger or smaller relative to the compared group was computed as the proportion of ellipse one that was smaller than ellipse two. Two SEAb were considered to be significantly different when more than 95% of the posterior estimates of one group were smaller than those of another group (Jackson et al. 2011).

To investigate for within‐species differences in the position of δ‐space among seasons and years, isotopic nestedness (Ines; Cucherousset and Villéger 2015) among all sampling periods for each species was calculated. Ines is the ratio between the area of the intersection of the two δ‐spaces being compared and the area of the smallest δ‐space of the two δ‐spaces being compared. It ranges from 0 when there is no overlap between δ‐spaces to 1 when the sampling period with the smallest δ‐space fills a subset of the sampling period with the largest δ‐space. To test the null hypothesis of two sampling periods' δ‐space not overlapping, sample labels were permuted between sampling periods being compared 1000 times. For each permutation, Ines was calculated and *p*‐values estimated as the proportion of times the observed overlap was greater than the permuted overlap.

Hypothesis 2b *Temporal variability in δ‐space of animals is driven by dynamic stable isotopic baselines*.

Spatial gradients of δ^13^C and δ^15^N values at the base of the food web are dynamic and respond quickly to fluctuating environmental variability (e.g., Espinasse et al. 2019; St John Glew et al. 2021). The trajectory of species δ‐spaces over time was investigated to test whether variability in the δ^13^C and δ^15^N blood values of the predators was an artefact of a shift at the baseline level. It was assumed that if all predators' δ^13^C and δ^15^N values followed similar trajectories over time this would suggest a change in the stable isotopic baselines rather than in the δ‐spaces. To track the movement of each species δ‐space across time, the Euclidean distance and bearing (0°–360°) between the centroid of the δ‐spaces was calculated between each year and separately for each season (Layman et al. 2007; Turner et al. 2010). To compare the species trajectories over time, the difference between the distance (ΔD) and angle (ΔA) moved by two species was tested to be significantly different from zero using a residual permutation procedure (RPP) following Turner et al. (2010), with adaptations to the published R code. This species‐by‐species analysis allowed for a critical evaluation of which individual species were affected by baseline changes, and which were not.

Hypothesis 3 *Resource partitioning within functional guilds is maintained over time*.

To assess whether resource partitioning was maintained through time within functional guilds (flying, diving), the Ines of each species δ‐space overlapping with all other species δ‐space was calculated for each sampling period (hereafter referred to as ‘inter‐species Ines’). Due to missing data for grey‐headed and light‐mantled albatrosses in summer 2016, inter‐species Ines was not calculated for this sampling period. Similarly, due to missing data for summer 2016 for both fur seals and autumn 2015 for Antarctic fur seal, inter‐species Ines was calculated separately for penguins for all sampling periods and then again including all diving species but excluding sampling periods autumn 2015 and summer 2016. To allow for comparisons, inter‐species Ines was calculated for 1000 δ‐spaces drawn from the posterior distribution for each species within each sampling period (R package *SIBER*; Jackson et al. 2011). The probability that the inter‐species Ines of a species was different between sampling periods was shown by calculating the proportional overlap of each sampling period posterior distribution of inter‐species Ines using the R package *overlap* (Pastore et al. 2022). A proportional overlap of 0 indicated a zero probability of sampling periods inter‐species Ines being similar and a value of 1 indicated that there was a 100% probability that the sampling period's inter‐species Ines were similar.

All values are reported as mean ± standard deviation.

## 3 Results

On average, 18 ± 4 and 17 ± 4 samples were collected per species during autumn and summer of each of the 3 years, respectively. In total, samples were collected from 115 wandering albatrosses, 97 grey‐headed albatrosses, 94 sooty albatrosses, 20 light‐mantled albatrosses, 117 northern giant petrels, 118 southern giant petrels, 111 king penguins, 53 gentoo penguins, 112 macaroni penguins, 111 eastern rockhopper penguins, 78 sub‐Antarctic fur seals and 91 Antarctic fur seals (Figure 2; Appendices S4 and S5).

**FIGURE 2:**
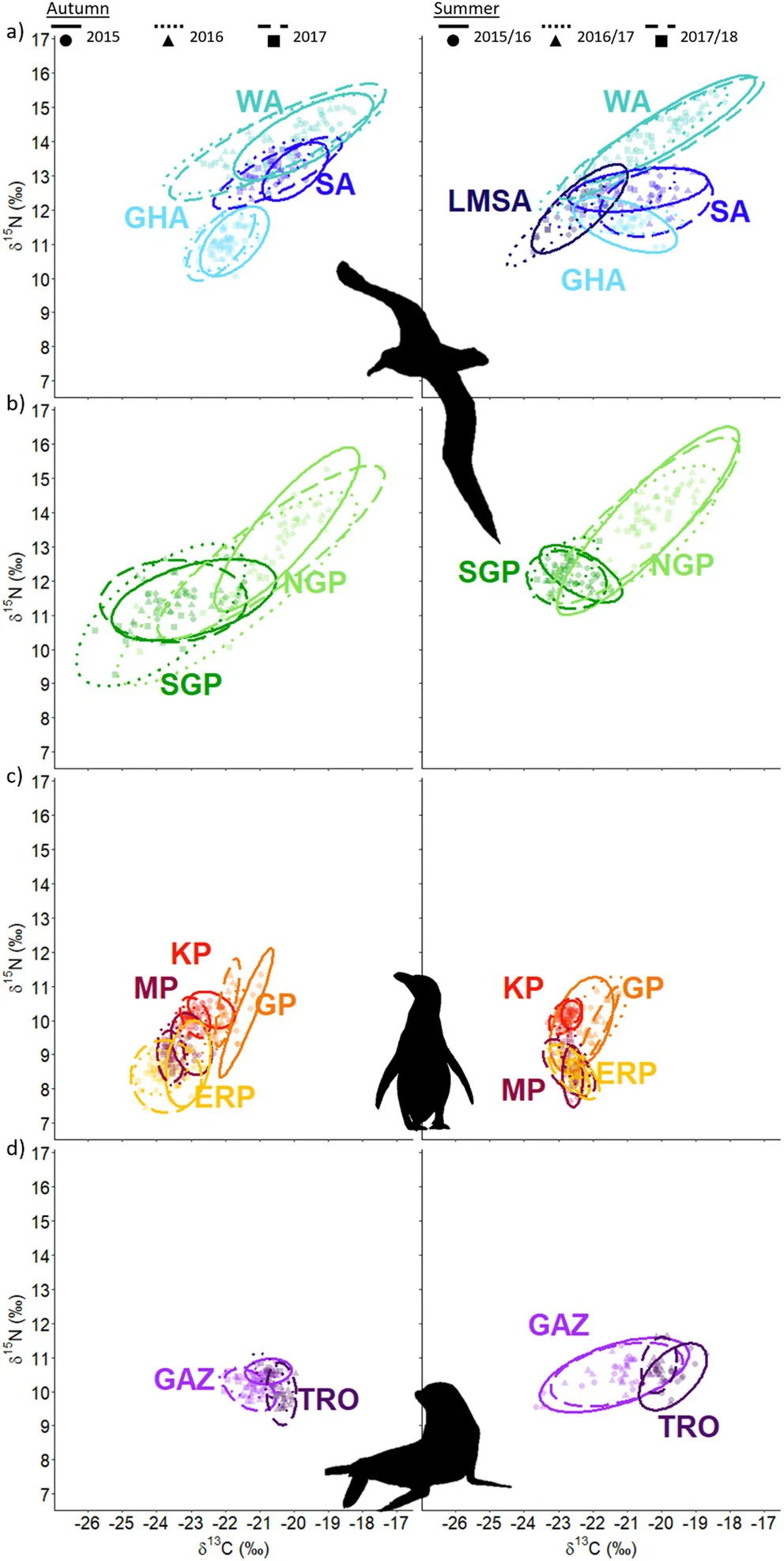
Standard ellipse areas at the 95% confidence interval calculated from the δ^13^C and δ^15^N blood values of (a) wandering (WA), grey‐headed (GHA), sooty (SA) and light‐mantled (LMSA) albatrosses, (b) northern (NGP) and southern (SGP) giant petrels, (c) king (KP), gentoo (GP), macaroni (MP) and eastern rockhopper (ERP) penguins, and (d) Antarctic (GAZ) and sub‐Antarctic (TRO) fur seals breeding on Marion Island over 3 years (2015–2017/2018) during autumn (left) and summer (right).

**Hypothesis 1.** *The δ‐space of animals is variable between seasons*.

The δ‐space of all 12 species' varied significantly between seasons in terms of either size (Figure 3a) or 2D location (Figure 3b). Consistent seasonal differences in δ‐space size over the years were only evident for Antarctic fur seals (larger δ‐space during summer) and southern giant petrels (larger δ‐space during autumn, Figure 3a), even though their δ‐space 2D position remained similar between seasons (Figures 2 and 3b). The remaining four flying species had similar δ‐space size but significantly different positions among seasons (Figures 2 and 3b). The δ‐space size and position of the four penguin species and sub‐Antarctic fur seal were variable across time with no evident pattern (Figures 2 and 3).

**FIGURE 3:**
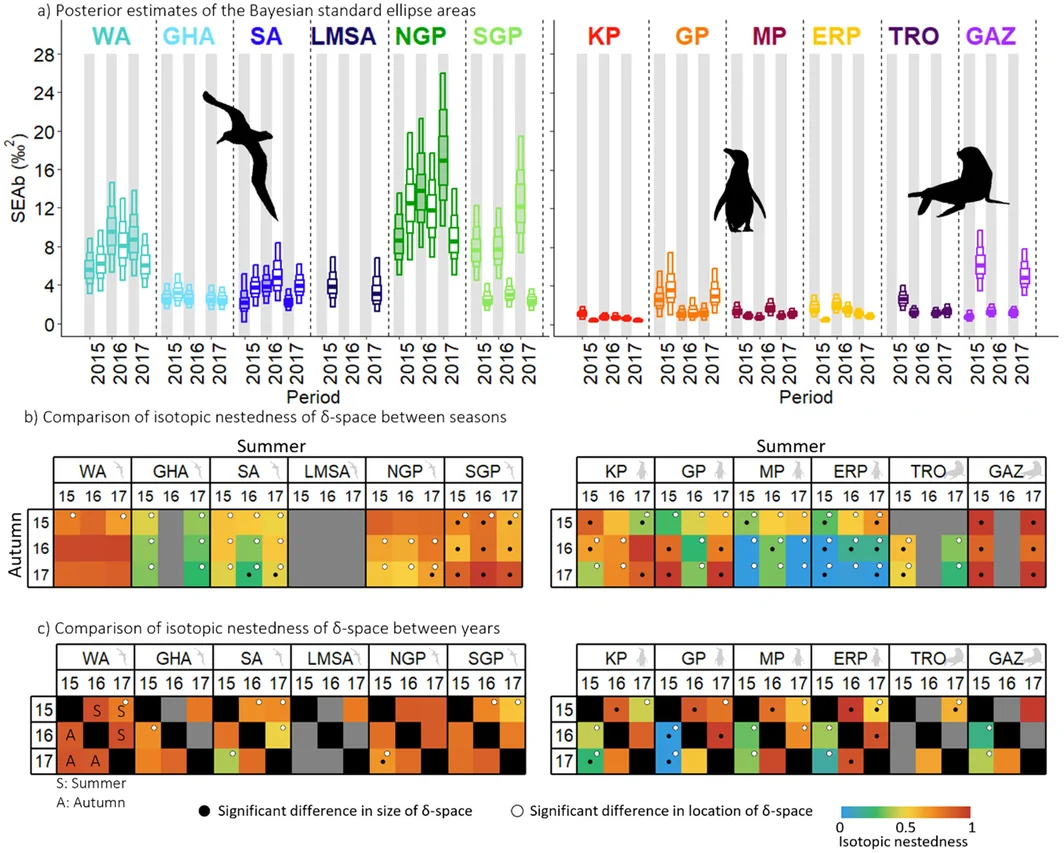
(a) Bayesian standard ellipse area (SEAb) at the 95% confidence interval with the 95%, 75% and 50% credible intervals of flying (left; wandering (WA), grey‐headed (GHA), sooty (SA) and light‐mantled (LMSA) albatrosses, northern (NGP) and southern (SGP) giant petrels) and diving species (right; king (KP), gentoo (GP), macaroni (MP) and eastern rockhopper (ERP) penguins and Antarctic (GAZ) and sub‐Antarctic (TRO) fur seals) on Marion Island during three autumns (shaded; 2015, 2016, 2017) and summers (not shaded; 2015/2016, 2016/2017, 2017/2018) over three consecutive years. Observed isotopic nestedness (b) among each species autumn (vertical) and summer (horizontal) δ‐space, and (c) year comparison among each species autumn (lower diagonal) and summer (upper diagonal) δ‐space, as indicated for WA. Black dots indicate significant differences between sizes of δ‐space (SEAb) and open dots indicate significant difference in location of δ‐space. Grey‐shaded areas indicate no data for comparisons.

**Hypothesis 2a.** *The δ‐space of animals is variable among years*.

Over the 3 years of the study period, the seasonal δ‐spaces of the flying species (albatrosses and giant petrels) were of similar size except for the δ‐space of northern giant petrel in autumn 2017 that was significantly larger than the δ‐space in autumn 2015 (Figure 3c). Similarly, the flying species seasonal δ‐spaces were similar in location among years with few exceptions (Figures 2 and 3c). Most noticeably, the sooty albatross occupied a significantly different δ‐space in all three summers. With regards to the diving species (penguins and fur seals), the area and location of their δ‐space varied significantly among years (Figure 3c). There was no clear trend in the variability between summers, however; for all diving species (excluding the sub‐Antarctic fur seals where data was not available) the species δ‐space in 2015 was significantly different to that of 2016 and 2017, whereas 2016 and 2017 were similar.

**Hypothesis 2b.** *δ‐space of animals is variable in time due to dynamic stable isotopic baselines*.

A clear pattern emerged over the 3 years with declining autumn δ^13^C and δ^15^N values observed for most species (Figure 4). The δ^13^C centroid values of wandering albatrosses, light‐mantled albatrosses, southern giant petrels, king penguins and sub‐Antarctic fur seals became progressively lower over the three summers (Figure 4a). The δ^15^N centroid values of wandering albatrosses and light‐mantled albatrosses also became progressively lower over the three summers, while those of sub‐Antarctic fur seals increased (Figure 4a). The centroid values and trajectory of eastern rockhopper and macaroni penguins were markedly similar, with δ^15^N decreasing over the three summers (Figure 4). There was no clear trend in summer δ‐space shift direction among the remaining species (sooty albatross, grey‐headed albatross, northern giant petrel and Antarctic fur seal) across years (Figure 4). However, the distance and direction to which most species' δ‐space centroids moved among summers and autumns were statistically similar (Appendices S6 and S7, respectively).

**FIGURE 4:**
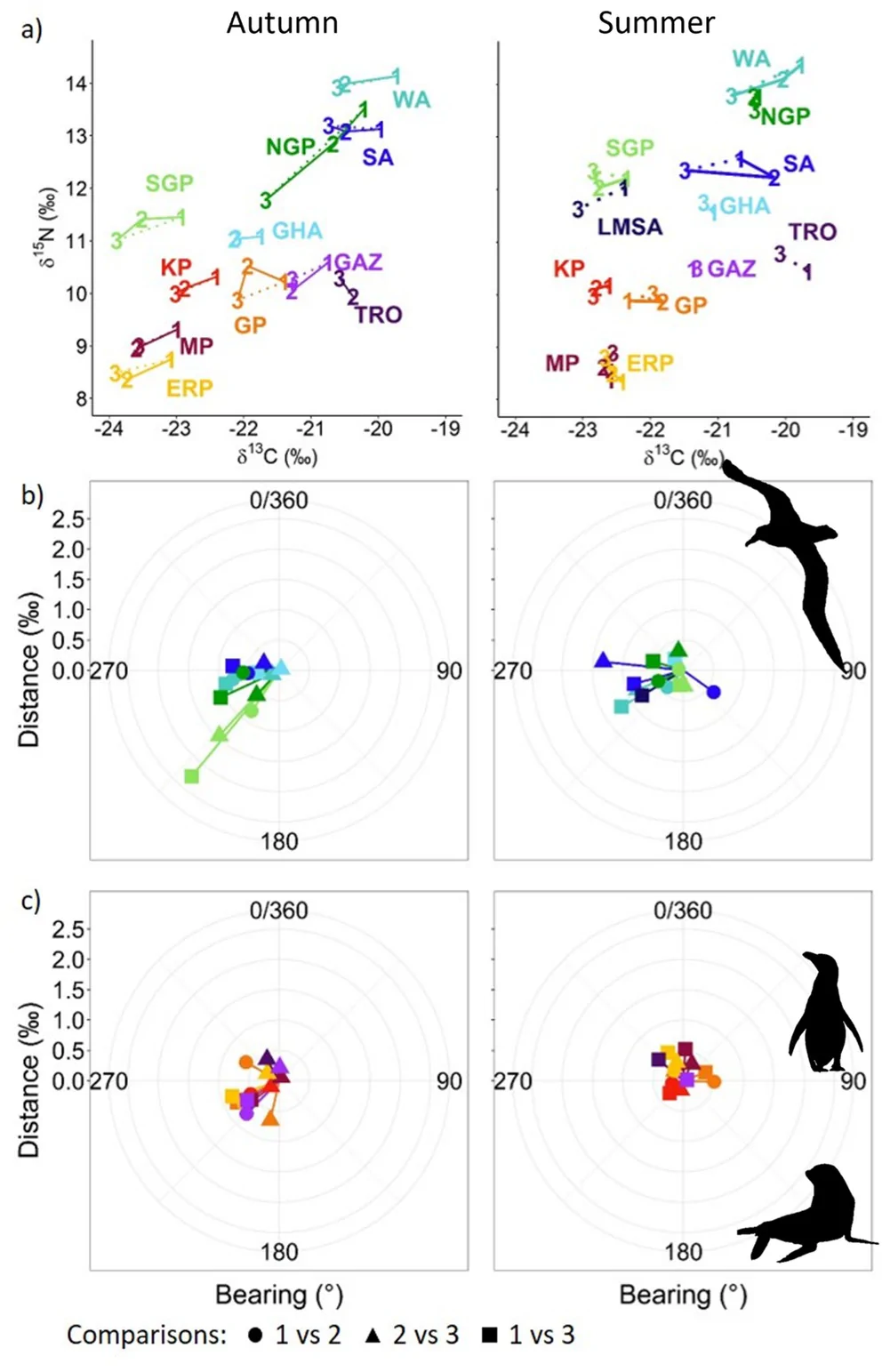
(a) Trajectories of stable isotopic niche centroids of all species except light‐mantled albatrosses over three autumns (left; 1: 2015, 2: 2016, 3: 2017) and all species over three summers (right, 1: 2015/2016, 2: 2016/2017, 3: 2017/2018), separated by (b) flying (wandering (WA), grey‐headed (GHA), sooty (SA), and light‐mantled (LMSA) albatrosses, northern (NGP) and southern (SGP) giant petrels) and (c) diving species (king (KP), gentoo (GP), macaroni (MP) and eastern rockhopper (ERP) penguins and Antarctic (GAZ) and sub‐Antarctic (TRO) fur seals).

**Hypothesis 3.** *Resource partitioning within functional guilds is maintained over time*.

Inter‐species Ines (i.e., resource partitioning in the 2D δ‐space) was more different between seasons than among years, however this trend was clearer for flying seabirds than among the penguin species (Figure 5). Greater resource partitioning (i.e., lower inter‐species Ines scores) was observed for wandering albatrosses and southern giant petrels during autumn, grey‐headed and sooty albatrosses during summer and similar levels of resource partitioning were observed between seasons for northern giant petrels (Figure 5b). However, within season, inter‐annual levels of inter‐species overlap remained more (or less) consistently similar (i.e., high proportional overlap of the resulting posterior distribution), meaning that resource partitioning in the 2D δ‐space was maintained among years (Figure 5b).

**FIGURE 5:**
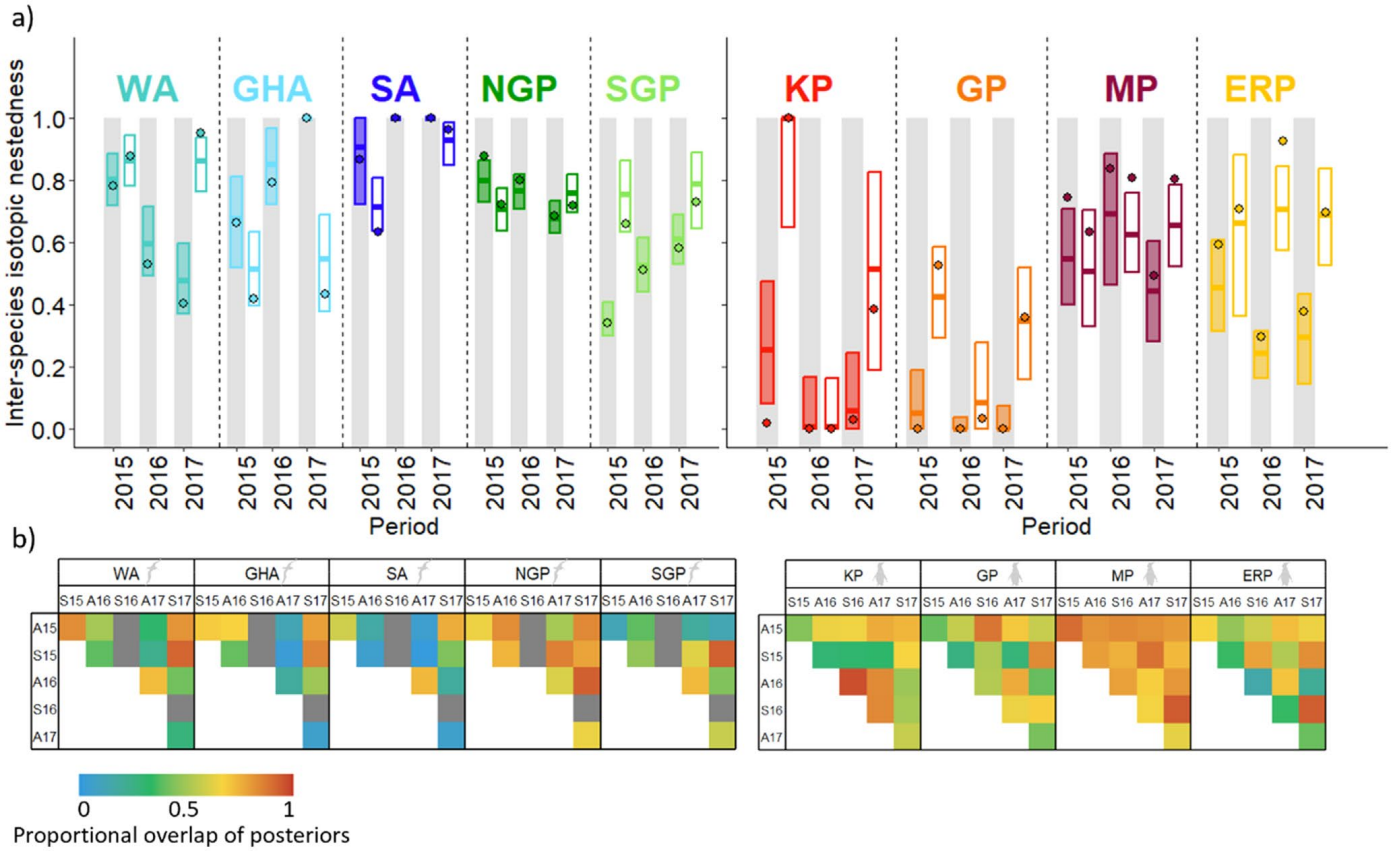
(a) Observed (dots) and 1000 posterior draws (mode and quantiles shown) of each species inter‐species stable isotopic nestedness (inter‐species Ines) per year and season of flying (left; wandering (WA), grey‐headed (GHA) and sooty (SA) albatrosses, northern (NGP) and southern (SGP) giant petrels) and penguin species (right; king (KP), gentoo (GP), macaroni (MP) and eastern rockhopper (ERP) penguins) on Marion Island during three autumns (A; shaded, 2015, 2016, 2017) and summers (S; not shaded 2015/2016, 2016/2017, 2017/2018) over three consecutive years. (b) Proportional overlap of the resulting posterior distribution per sampling period and species indicating probability of inter‐species Ines being completely overlapping (value of 1) or completely segregated (value of 0). Grey‐shaded areas indicate no data for comparisons.

With regards to the penguins, only eastern rockhopper penguins were observed to have a clear seasonal difference in resource partitioning (i.e., inter‐species Ines, Figure 5a). The level of resources partitioning for king, macaroni and gentoo penguins were consistent throughout sampling periods with the exception of the 2015 and 2017 summers, where gentoo and king penguins' inter‐species Ines values indicated high levels of resource partitioning (Figure 5b). Interestingly, the comparison of inter‐species Ines of penguins and fur seals, for sampling periods where data from fur seals were available, showed higher levels of resource partitioning and clearer seasonal patterns (Figure 6). However, as observed with the flying species Ines, inter‐annual levels of inter‐species remained more (or less) consistently similar (i.e., high proportional overlap of the resulting posterior distribution) within season, meaning that resource partitioning in the 2D δ‐space was maintained among years (Figure 6b).

**FIGURE 6:**
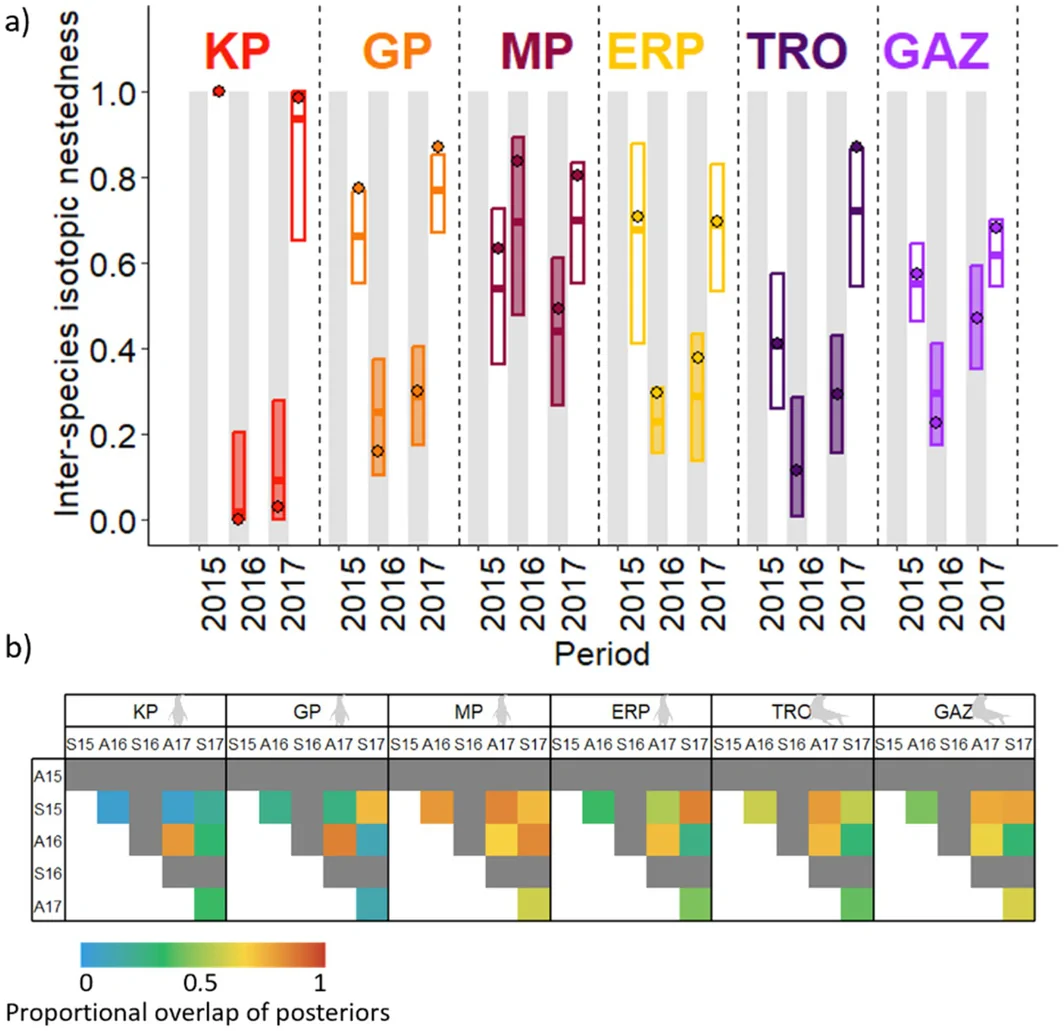
(a) Observed (dots) and 1000 posterior draws (mode and quantiles shown) of each diving species (king (KP), gentoo (GP), macaroni (MP) and eastern rockhopper (ERP) penguins and sub‐Antarctic (TRO) and Antarctic (GAZ) fur seals) inter‐species stable isotopic nestedness (inter‐species Ines) per year and season where data was available on Marion Island during two autumns (A; shaded, 2016 and 2017) and summers (S; not shaded 2015/2016 and 2017/2018). (b) Proportional overlap of the resulting posterior distribution per sampling period and species indicating probability of inter‐species Ines being completely overlapping (value of 1) or completely segregated (value of 0). Grey‐shaded areas indicate no data for comparisons.

## 4 Discussion

Using δ^13^C and δ^15^N whole blood compositions, we showed that there was significant variation in the seasonal and annual δ‐space of marine predators within different taxonomic groups and functional guilds. Despite this marked variability, resource partitioning within guilds was consistently maintained through time. Our results highlight two major factors driving the foraging behaviour of these animals. Firstly, most of these species have foraging behaviours that are either sufficiently flexible or general to cope with resources that are variable in space and time (Dehnhard et al. 2020; Friscourt et al. 2026; Riotte‐Lambert and Matthiopoulos 2019). Secondly, whether due to historic or present competition for resources (Connell 1980), these predators have foraging strategies that minimise interspecific overlap in trophic and/or spatial resource use.

**Hypothesis 1.** *The δ‐space of animals is variable between seasons*.

The δ‐space of the 12 predator species varied more between autumn and summer than among years, in terms of area, 2D location or both. This seasonal signal suggests that foraging behaviour is primarily shaped by life‐history‐related constraints such as breeding stage and offspring provisioning, rather than by inter‐annual environmental variability alone. For example, the δ^13^C values of grey‐headed and sooty albatrosses correlate to what is known about their seasonal foraging behaviour. While incubating (summer), the two albatross species forage primarily north of Marion Island, travelling farther than during chick‐rearing (autumn; Carpenter‐Kling, Reisinger, et al. 2020). However, while rearing a chick, grey‐headed albatross primarily forage south of the island (Nel et al. 2001), consuming Antarctic prey (Connan et al. 2014; Richoux et al. 2010). In constrast, sooty albatross forage north of the island throughout the chick‐rearing period, consuming sub‐tropical prey (Connan et al. 2014; Schoombie et al. 2017). The lower δ^15^N blood values of grey‐headed albatross during autumn may be due to a switch in diet composition or feeding of a chick: grey‐headed albatross parents provide higher trophic level and more nutritious prey to their offspring while digesting lower trophic level and less nutritious prey themselves (Richoux et al. 2010). In addition, the significantly larger δ‐space of Antarctic fur seal during autumn compared to summer can also be explained by what is known about their foraging behaviour. During summer, the foraging distribution of female Antarctic fur seals is constrained by the need to return to Marion Island and provide for their pup as well as potentially reducing inter‐ and intra‐specific competition for resources with other similarly constrained breeding seals and seabirds (Friscourt et al. 2026; Wege et al. 2019). Once pups are weaned in April the foraging distribution of female Antarctic fur seals significantly increases (Wege et al. 2019). Individual females have been shown to use two distinct wintering foraging areas, either travelling north to the Sub‐Antarctic Front to forage on mesopelagic fish and cephalopods or southward of the Antarctic Polar Front to forage on lower trophic‐level prey, most likely Antarctic krill ( *Euphausia superba* ; Arthur et al. 2015; Walters et al. 2020). This dichotomy of foraging behaviours during winter would explain the much larger δ‐space we observed for Antarctic fur seal during autumn compared to during summer. Seasonal segregation of foraging areas has also been documented for the two *Eudyptes* penguin species (macaroni and eastern rockhopper penguins; Whitehead et al. 2016) and sooty albatross (Carpenter‐Kling, Reisinger, et al. 2020; Schoombie et al. 2017, 2022), supporting the interpretation that seasonal changes in δ‐space primarily reflect predictable shifts in foraging constraints rather than stochastic resource variation.

**Hypothesis 2a.** *The δ‐space of animals is variable among years*.

Although some inter‐annual variability in δ‐space position was evident for flying seabirds, particularly sooty albatrosses and southern giant petrels during summer, inter‐annual variability was substantially greater for diving predators. Whereas the area of flying seabird δ‐spaces was largely consistent within seasons among years (with the exception of northern giant petrels between autumn 2015 and 2017), the area or 2D location of the diving species' δ‐space was nearly never consistent within seasons and between years (except for Antarctic fur seal summer and sub‐Antarctic fur seal autumn δ‐spaces). We expected all species δ‐spaces to exhibit variability among years as a response to inter‐annual change in prey availability and subsequently prey switching by predators to temporally available prey (Carpenter‐Kling, Handley, et al. 2019; Reisinger et al. 2018) or to foraging in more profitable habitats (Carpenter‐Kling, Reisinger, et al. 2020; Kappes et al. 2010). The pronounced variability in the δ‐space of diving species is most probably an outcome of their much more restricted foraging ranges and inability to travel vast distances quickly compared to flying seabirds and thus are more influenced by inter‐annual changes in prey availability. The consistency of the Antarctic fur seal summer δ‐space among years is likely due to their high interannual site fidelity (Arthur et al. 2015), and the lack thereof during autumn might be due to sampling biases of females which prefer to forage north or south, as individuals have also been observed to show high interannual site fidelity during the non‐breeding period (Arthur et al. 2015; Friscourt et al. 2026; Walters et al. 2020).

The predator‐specific generalist or specialist foraging habitat preference or dietary composition may explain within guild (i.e., flying vs. diving) variability in the dynamics of the species' δ‐spaces over time. For example, the wandering and sooty albatrosses are known foraging habitat generalist (Carpenter‐Kling, Reisinger, et al. 2020), however, wandering albatrosses are assumed to be dietary specialists (low variability in dietary composition; Adams and Klages 1987; Xavier et al. 2003) and sooty albatrosses are assumed to be dietary generalist (high temporal variability in dietary composition, Banda 2019). In addition, wandering albatrosses have been described as ‘wandering nomads’, covering vast areas and visiting different types of foraging habitats, whereas the sooty albatrosses have been described as ‘faithful nomads’, foraging within a limited geographical area but in various foraging habitats, switching annually to more productive oceanographic features (Carpenter‐Kling, Reisinger, et al. 2020). Thus, the large geographic range and narrow trophic/dietary range of wandering albatrosses may explain the species consistancy in δ‐space over time (Orgeret et al. 2021). And contrastingly, the broad trophic/dietary range and ability to switch between foraging habitats may explain the inter‐annual summer δ‐space variability for sooty albatrosses.

All species of penguins exhibited inter‐annual changes within seasons in their δ‐space, albeit the area or 2D location or both. Due to the similarities in the gentoo, macaroni and eastern rockhopper penguins' diet composition (Brown and Klages 1987; Carpenter‐Kling, Handley, et al. 2019; Dakwa et al. 2021) and relatively large populations of macaroni and eastern rockhopper penguins breeding on Marion Island (Crawford et al. 2009), it has been proposed that these three penguin species compete for resources during summer. Although the gentoo penguin is a well‐described foraging and dietary generalist (Carpenter‐Kling, Handley, et al. 2019; Lescroël et al. 2004), all three species have been reported to exhibit a generalist dietary composition (Carpenter‐Kling, Handley, et al. 2019; Dakwa et al. 2021; Horswill et al. 2016). However, unlike gentoo penguins, macaroni and eastern rockhopper penguins do not have the additional energy demands of a chick during summer (when samples from these species were collected) and thus, are likely not as restricted as breeding macaroni and eastern rockhopper penguins to forage for energy‐rich food, resulting in the observed much larger δ‐space and changes in the δ‐space 2D location.

**Hypothesis 2b.** *δ‐space of animals is variable in time due to dynamic stable isotopic baselines*.

The congruent declines in the δ^13^C and δ^15^N blood values of multiple species suggest a possible shift in the stable isotopic gradients at the base of the food web, as found by Ramos et al. (2020), Mestre et al. (2020), Tarroux et al. (2018), Friscourt et al. (2024) and Séon et al. (2025). However, only the species that are known to forage at (king penguin; Bost et al. 2015; Pistorius et al. 2017) or south of (macaroni and eastern rockhopper penguins autumn only; southern giant petrel and wandering, light‐mantled and grey‐headed albatrosses; Nel et al. 2001, 2002; Reisinger et al. 2020; Carpenter‐Kling, Reisinger, et al. 2020; Whitehead et al. 2016) the Antarctic Polar Front exhibited progressively lower δ^13^C values. This coincided with the Antarctic Polar Front moving closer to Marion Island as the years progressed (Carpenter‐Kling, Reisinger, et al. 2020). Because travelling time to waters south of the Antarctic Polar Front should be inversely correlated with the time spent foraging south of the front, the movement of the front is likely a driver behind the progressively more negative δ^13^C values of southern giant petrels and wandering, light‐mantled and grey‐headed albatrosses as well as autumn δ^13^C values of macaroni and eastern rockhopper penguins. Further evidence to support this inference is that northern giant petrels and sooty albatrosses, which forage almost exclusively north of Marion Island during summer (Reisinger et al. 2020; Schoombie et al. 2017), did not share this declining pattern in their summer δ^13^C blood values. Instead, sooty albatrosses' summer δ^13^C blood values followed the movement of core foraging areas found by Carpenter‐Kling, Reisinger, et al. (2020), that is, the key foraging area of the sooty albatross was found closer to Marion Island in 2015/2016 and 2017/2018, and further north in 2016/2017.

Although we argue that there was no shift in the baseline stable isotopic gradients, this confounding factor in the stable isotopic composition of marine predator tissue values is rarely considered and, if it is considered, it has been shown to have an influence on the stable isotopic composition for animals' tissues (e.g., Friscourt et al. 2024). Spatial gradients of δ^13^C and δ^15^N values of organisms at the base of the food web are dynamic and respond quickly to fluctuating environmental variability (e.g., Magozzi et al. 2017; Walters et al. 2020). Interestingly, the monthly isoscapes produced by Magozzi et al. (2017) showed a stronger latitudinal gradient of phytoplankton δ^13^C values during summer, with higher and lower values evident between South Africa and Antarctica, respectively, compared to autumn. However, the δ^13^C blood values of most predators decreased between summer and autumn, even though the regional stable isotopic baseline increased. Therefore, although temporal variability in baseline stable isotopic gradients most likely introduces some variance into the stable isotopic composition of predator tissues (Carpenter‐Kling, Pistorius, et al. 2019, 2020), it seems unlikely to be driving the patterns we observed. Future investigation should combine fine‐scale logging devices (such as GPS loggers) with bulk stable isotope analysis (e.g., Carpenter‐Kling, Pistorius, et al. 2019, 2020; Trueman et al. 2019) or compound‐specific isotope analysis of amino acids in blood (e.g., Friscourt et al. 2024) to further elucidate possible influences of temporal changes in the baseline stable isotopic gradients on marine predator tissue composition.

**Hypothesis 3.** *Resource partitioning within functional guilds is maintained over time*.

The maintenance of resource partitioning among the 12 predators suggests that these predators forage in a resource‐limited environment (either currently or historically) and they partition food resources to limit inter‐specific competition (Jaeger et al. 2013; Pickett et al. 2018). Although prey distribution within this area of the ocean is characteristically variable in space and time (Trathan et al. 2007; Lévy et al. 2015), the consistency of seasonal partitioning suggests a degree of predictability in resource availability. For example, Dehnhard et al. (2020), found little evidence of resource partitioning among three sympatric fulmarine species breeding in Antarctica across the annual cycle. The authors explained that this lack of partitioning among the three closely related species was linked to the highly productive but highly spatio‐temporally variable (i.e., unpredictable) nature of their environment. They hypothesised that these birds' generalist foraging behaviour evolved to maximise prey encounters in this ‘unpredictable’ environment, even though it most likely increased the potential for competition within and among sympatric species. Furthermore, some studies suggest that due to the super‐abundance of Antarctic krill in Antarctic waters, their predators may not have to partition their diet and foraging habitats in order to avoid competitive exclusion (Croxall et al. 1997; Jaeger et al. 2013). The clear segregation among predators' δ‐space in our study indicates that resources (presently or historically) are limited, as found for other sub‐Antarctic islands (Bodey et al. 2014; Cherel et al. 2007; Jaeger et al. 2010; Moreno et al. 2016). These patterns may reflect both competitive processes and evolutionary specialisation, which are not mutually exclusive.

### 4.1 Study Caveats and Future Research Priorities

While our findings provide valuable insights into temporal patterns of resource use and partitioning among sympatric marine predators, they should be interpreted in light of several methodological and ecological caveats that also highlight priorities for future research.

Our study uses the δ‐space of marine predator species as a proxy for their trophic niche, with δ^13^C broadly indicating spatial distribution and δ^15^N indicating trophic position within an ecosystem (Newsome et al. 2007; Petalas et al. 2024). This approach has allowed us to test three hypotheses (Figure 1) of how these sympatric marine species use their environments over time and potentially facilitate their co‐existence by partitioning resources. However, there are limitations to this approach that must be considered. Although the δ^15^N composition of animal tissues indicates its trophic position within an ecosystem (Newsome et al. 2007), it does not reveal potential differences in specific prey species and/or taxa composition of different predators' diets. Furthermore, here we only considered horizontal gradients or isoscapes in δ^15^N and δ^13^C but stable isotope values are also known to vary with depth (Choy et al. 2015; Hannides et al. 2013; Romero‐Romero et al. 2019) and between inshore benthic habitats and offshore pelagic habitats (Carpenter‐Kling, Pistorius, et al. 2020; Somes et al. 2010; Takai et al. 2000). With these limitations in mind, we discuss whether and to what extent our results support our hypotheses, while recognising that the use of δ‐space represents a proxy, or ‘broad‐brush approach’, for understanding resource partitioning among sympatric predators over time. Such resource partitioning is inherently driven by a complex interplay of multidimensional factors. Furthermore, this study provides evidence that spatial and trophic resource use of sympatric seabirds and fur seals is influenced by foraging behavioural changes associated with different life history stages, changes in the environment (resource distribution or stable isotopic baselines) and maintaining inter‐specific resource partitioning. However, resource use of seabirds and seals is influenced by other intra‐population factors such as sexual segregation (e.g., Nel et al. 2001; Orgeret et al. 2020; Resinger et al. 2020), age‐related factors (e.g., Votier et al. 2017) and individual specialisation (e.g., Ceia and Ramos 2015; Collet et al. 2025; Friscourt et al. 2026). The main aim of this study was to investigate intra‐species and inter‐species changes in stable isotopic niches over time and therefore species‐specific samples were grouped into autumns and summers of 3 years. This resulted in per‐season and per‐year sample sizes ranging from 6 to 20 samples, which precluded our ability to fully capture intra‐population variation associated with intrinsic factors such as sex, age and individual differences.

Finally, the relatively small sample sizes for some species for some sampling sessions may have underrepresented intra‐population variability and individual specialisation, meaning that the sampled individuals may not have captured the full range of foraging strategies within each species. Although Bayesian standard ellipses are generally robust to small sample sizes, niche estimates may still be sensitive especially for groups with *n* < 10 (Jackson et al. 2011). Therefore, differences in stable isotopic niche size among groups with small sample sizes should be interpreted with caution. Future studies incorporating larger sample sizes would help to better characterise intra‐population variation and provide more robust estimates of the stable isotopic niche of animals.

## 5 Conclusions

According to Hutchinson (1957), animals have a fundamental niche, an n‐dimensional space a species occupies in the absence of competitors. However, this space is reduced in the presence of competitors to the species ‘realised niche’; this can influence numerous variables or dimensions of animal resource use such as where and when to breed, sleep or eat. Here, we studied resource partitioning using the carbon and nitrogen stable isotopic composition of 12 sympatric marine predators within two functional guilds: flying and diving. Using this 3‐dimensional data, geographical (δ^13^C, although limited), trophic (δ^15^N) and temporal (δ^15^N and δ^13^C compositions from two seasons over 3 years), we have started unravelling how these sympatric predators and predators elsewhere, foraging for similar prey, are able to coexist. However, our study did not consider other variables that may influence this apparent dynamic resource partitioning we observed, such as how time can influence this partitioning in other ways. For example, the large populations (Crawford et al. 2006) and similar dietary composition (Brown and Klages 1987; Dakwa et al. 2021) of the two *Eudyptes* penguins on Marion Island suggest that these species may compete for resources. However, it has been speculated that their coexistence is facilitated by macaroni penguins initiating breeding 2–3 weeks prior to eastern rockhopper penguins (Crawford et al. 2006), which segregates energy demand by offspring, and by the ability of the former to dive deeper than the latter, thereby segregating the species spatially within the water column (Whitehead 2017). However, even without trying to incorporate the numerous variables and dimensions that may influence these predators' ‘realised’ trophic niches, we show that all 12 species have dynamic stable isotopic niches, likely driven by variability in prey availability and foraging constraints associated with different life history stages. Importantly, we demonstrate that resource partitioning within functional guilds is maintained despite the evident dynamic foraging behaviour of the predators. This is consistent with classical niche theory (Hutchinson 1957; Pianka 1974; Schoener 1974), which predicts that animals will adjust their foraging behaviour during periods of relative food scarcity to decrease interspecific competition. However, the consistent partitioning of resources among predators over all 3 years indicates that the resources of these predators are either historically or presently limited and their coexistence is promoted by partitioning along the trophic axes of their ecological niches. This has important consequences for studies which aim to predict distributional changes of species in relation to climate change (e.g., Reisinger et al. 2022). Indeed, most of these investigations only consider the environmental factors/axes which influence a species' distribution and rarely incorporate the influence of interspecific interactions.

## Author Contributions

**T. Carpenter‐Kling:** conceptualization (equal), data curation (equal), formal analysis (equal), writing – original draft (equal). **M. Connan:** conceptualization (equal), data curation (equal), methodology (equal), supervision (equal), writing – review and editing (equal). **R. R. Reisinger:** conceptualization (equal), formal analysis (equal), supervision (equal), writing – review and editing (equal). **A. B. Makhado:** conceptualization (equal), funding acquisition (equal), project administration (equal), writing – review and editing (equal). **P. G. Ryan:** conceptualization (equal), funding acquisition (equal), methodology (equal), writing – review and editing (equal). **P. A. Pistorius:** conceptualization (equal), data curation (equal), funding acquisition (equal), project administration (equal), supervision (equal), writing – review and editing (equal).

## Funding

This work was supported by South Africa's National Research Foundation (NRF), through its South African National Antarctic Programme, as a grant to Pierre Pistorius (grant number SNA093071), and Ryan Reisinger was the recipient of a NRF postdoctoral grant (SANCOR grant 94916).

## Ethics Statement

The project received ethics clearance from the Nelson Mandela University Animal Ethics Committee (A14‐SCI‐ZOO‐012).

## Conflicts of Interest

The authors declare no conflicts of interest.

## Supporting information


**Data S1:** ece374579‐sup‐0001‐Supinfo1.docx.

## Data Availability

The data supporting the findings of this study have been deposited in the Dryad Digital Repository and are accessible via the permanent DOI: https://datadryad.org/dataset/doi:10.5061/dryad.pzgmsbd2v.
