# Supplementary material for: Dynamic Resource Partitioning in a Marine Predator Community
Source: Ecol Evol. 2026 Nov 2;16(11):e74579. doi: 10.1002/ece3.74579 (PMC13727373; doi:10.1002/ece3.74579)
Supplement: Supplementary file 1 [file ECE3-16-e74579-s001.docx]

**Supporting Information**

**Appendix S1.** Simplified graphic illustration depicting overlap of dominant prey in the diet of wandering (WA; Cooper et al., 1992), grey-headed, sooty and light-mantled albatrosses (GHA, SA and LMSA; Banda, 2019; Connan et al., 2014,) albatrosses, northern and southern giant petrels (NGP and SGP; Hunter & Brooke 1992), king (KP; Brown et al., 1990), gentoo (GP; Carpenter-Kling et al. 2019), macaroni and eastern rockhopper penguins (MP and ERP; Dakwa et al., 2021) and sub-Antarctic and Antarctic fur seals breeding (TRO and GAZ; Reisinger et al., 2018) on Marion Island.

**Appendix S2**. Approximate incubation (INC), brooding (BRO), chick rearing (CHI), chick créching (CRE), pupping (PUP) and lactation (LAC) periods and the two periods when individuals (number sampled next to period) of wandering (WA; Jones et al., 2016), grey-headed (GHA; FitzPatrick unpubl. data; Tickell & Pinder, 1975), sooty and light-mantled (SA and LMSA; Berruti, 1979; Schoombie et al., 2016) albatrosses, northern and southern giant petrels (NGP and SGP; Cooper et al., 2001), king (KP; du Plessis et al., 1994; Charrassin & Bost 2001), gentoo (GP; Crawford et al., 2003), macaroni and eastern rockhopper (MP and ERP; Crawford et al., 2006) penguins and sub-Antarctic and Antarctic (TRO and GAZ; Kerley 1983; Hofmeyr et al., 2007) fur seals breeding on Marion Island were sampled over three consecutive years (1: autumn 2015 2: summer 2015/16 and autumn 2016, 3: summer 2016/17 and autumn 2017 and 4: summer 2017/18).

**Appendix S3**

The whole blood was then dried at 50°C for 48 hrs before being powdered using a mortar and pestle. The isotopic composition of carbon and nitrogen in aliquots (~0.4 mg) of samples were determined by combusting samples in a Flash 2000 organic elemental spectrometer via a Conflo IV gas control unit (Thermo Scientific, Germany). All samples were processed at the Stable Light Isotope Unit at the University of Cape Town, South Africa. Replicate measurements of internal laboratory standards indicated minimal standard deviations within and among runs (Merck gel: SD_δ13C_ = 0.2 ‰, SD_δ15N_ < 0.1 ‰; valine: SD_δ13C_ < 0.2 ‰, SD_δ15N_ = 0.1 ‰; seal bone:SD_δ13C_ < 0.2 ‰, SD_δ15N_ < 0.1 ‰). All in-house standards were calibrated against International Atomic Energy Agency standards. Results are presented in the usual δ notation relative to Vienna PeeDee Belemnite and atmospheric N_2_ for δ^13^C and δ^15^N, respectively.

High lipid contents within tissue samples, detected by a C:N mass ratio > 3.5 in tissues for aquatic animals (Post et al., 2007), can lead to low δ^13^C values which may lead to misinterpretation of δ^13^C tissue values (Cherel, Hobson & Weimerskirch, 2005; DeNiro & Epstein 1977;). Most whole blood samples collected had a C:N mass ratio within normal ranges (Table 1) except blood samples collected from MP and ERP during autumn and LMSA during summer 2015/16 (C:N > 3.5). Samples from MP and ERP were collected from moulting birds during autumn (Appendix S2). During this time, the MP and ERP are fasting, using their endogenous nutrient stores for nutrition (Cherel, Hobson, Bailleul et al., 2005). Although the effects of this metabolic process is minimal on δ^15^N, the process leads to an increase in circulating lipids within the blood (Cherel, Hobson, Bailleul et al., 2005). An effect of lipids on δ^13^C was evident for both species due to the significant increase of their C:N mass ratios of their blood between summer and autumn (paired t-test: MP: t = -10.58, df = 72.75, p < 0.01; ERP: t = -8.72, df = 72.46, p < 0.01). This was corrected by mathematically normalizing blood δ^13^C values following Post et al., (2007).

**Appendix S4****.** δ^13^C and δ^15^N whole blood values and corresponding C:N ratios of 12 marine predators breeding on Marion Island over three years (2015 – 2017) during autumn and summer. “-” indicates no data. Mean ± SD (range) values are given.

**Appendix S5.** a) δ^13^C and b) δ^15^N blood values of flying (left; wandering (WA), grey-headed (GHA), sooty (SA) and light-mantled (LMSA) albatrosses, northern (NGP) and southern (SGP) giant petrels) and diving species (right; king (KP), gentoo (GP), macaroni (MP) and eastern rockhopper (ERP) penguins and sub-Antarctic (TRO) and Antarctic (GAZ) fur seals) breeding on Marion Island during three autumns (shaded, 2015, 2016, 2017) and three summers (not shaded, 2015/16, 2016/17, 2017/18). Values presented are median, 25% and 75% quartiles and range.

**Appendix S6**. Difference in the path length (upper right diagonal) and direction (lower left diagonal) of isotopic shifts between three consecutive summers: a) 2015/16 vs 2016/17, b) 2016/17 vs 2017/18 and c) 2015/16 vs 2017/18 of 12 marine predator species (wandering (WA), grey-headed (GHA), sooty (SA) and light-mantled (LMSA) albatrosses, northern (NGP) and southern (SGP) giant petrels, king (KP), gentoo (GP), macaroni (MP) and eastern rockhopper (ERP) penguins and Antarctic (GAZ) and sub-Antarctic (TRO) fur seals) and empirical p-values estimated from permutations procedures (Turner et al. 2010). Significant differences are shaded. “-” indicates no data for comparisons.

**Appendix S7**. Difference in the path length (upper right diagonal) and direction (lower left diagonal) of isotopic shifts between three consecutive autumns: a) 2015 vs 2016, b) 2016 vs 2017 and c) 2015 vs 2017 of 12 marine predator species (wandering (WA), grey-headed (GHA), sooty (SA) and light-mantled (LMSA) albatrosses, northern (NGP) and southern (SGP) giant petrels, king (KP), gentoo (GP), macaroni (MP) and eastern rockhopper (ERP) penguins and Antarctic (GAZ) and sub-Antarctic (TRO) fur seals) and empirical p-values estimated from permutation procedures in brackets (Turner et al. 2010). Significant differences are shaded. “-” indicates no data for comparisons.

**Cited literature**

Banda S (2019) Trophic ecology of the Sooty Albatross Phoebetria fusca at sub-Antarctic Marion Island. BSc Honours Thesis, Nelson Mandela University, South Africa

Berruti A (1979) The breeding biologies of the sooty albatrosses *Phoebetria fusca* and *P. palpebrata*. Emu 79:161–175.

Brown CR, Klages NTW, Adams NJ (1990) Short and medium-term variation in the diets of penguins at Marion Island. S Afr T Nav Antarkt 20:13 – 20.

Carpenter-Kling T, Handley JM, Connan M, Crawford RJM, Makhado AB, Dyer BM, Froneman W, Lamont T, Wolfaardt AC, Landman M, Sigqala M, Pistorius PA (2019) Gentoo penguins as sentinels of climate change at the sub-Antarctic Prince Edward Archipelago, Southern Ocean. Ecol Indic 101:163–172.

Charrassin JB, Bost CA (2001) Utilisation of the oceanic habitat by king penguins over the annual cycle. Mar Ecol Prog Ser 221:285–297.

Cherel Y, Hobson KA, Weimerskirch H (2005a) Using stable isotopes to study resource acquisition and allocation in procellariiform seabirds. Oecologia 145:533–540.

Cherel Y, Hobson KA, Bailleul F, Groscolas R (2005b) Nutrition, physiology, and stable isotopes: New information from fasting and moulting penguins. Ecology 86:2881–2888.

Connan M, McQuaid CD, Bonnevie BT, Smale MJ, Cherel Y (2014) Combined stomach content, lipid and stable isotope analyses reveal spatial and trophic partitioning among three sympatric albatrosses from the Southern Ocean. Mar Ecol Prog Ser 497:259–272.

Cooper J, Henley SR, Klages NTW (1992) The diet of the wandering albatross *Diomedea exulans* at Subantarctic Marion Island. Polar Biol 12:477 – 484.

Crawford RJM, Cooper J, Du Toit M, Greyling MD, Hanise B, Holness CL, Keith DG, Nel JL, Petersen SL, Spencer K, Tshingana D, Wolfaardt AC (2003) Population and breeding of the gentoo penguin *Pygoscelis papua* at Marion Island, 1994/95-2002/03. Afr J Mar Sci 25:463–474.

Crawford RJM, Dyer BM, Cooper J, Underhill LG (2006) Breeding numbers and success of *Eudyptes* penguins at Marion Island, and the influence of mass and time of arrival of adults. CCAMLR Sci 13:175–190.

Dakwa F, Ryan P, Dyer B, Crawford R, Pistorius P, Makhado A (2021) Long-term variation in the breeding diets of macaroni and eastern rockhopper penguins at Marion Island (1994–2018). Afr J Mar Sci 43:187–199.

DeNiro MJ, Epstein S (1977) Mechanism of carbon isotope fractionation associated with lipid synthesis. Science 197:261–263.

du Plessis CJ, Van Heezik YM, Seddon PJ (1994) Timing of king penguin breeding at Marion Island. Emu 94:216–219.

Hunter S, Brooke ML (1992) The diet of giant petrels *Macronectes* spp. at Marion Island, Southern Indian Ocean. Colon Waterbirds 15:56–65.

Hofmeyr GJG, Bester MN, Pistorius PA, Mulaudzi TW, De Bruyn PJN, Ramunasi JA, Tshithabane HN, McIntyre T, Radzilani PM (2007) Median pupping date, pup mortality and sex ratio of fur seals at Marion Island. Afr J Wildl Res 37:1–8.

Jones MGW, Dilley BJ, Hagens QA, Louw H, Mertz EM, Visser P, Ryan PG (2016) Wandering albatross *Diomedea exulans* breeding phenology at Marion Island. Polar Biol 40:1139–1148.

Kerley GIH (1983) Comparison of seasonal haul-out patterns of fur seals *Arctocephalus tropicalis* and *A. gazella* on subantarctic Marion Island. S Afr J Wildl Res 13:71–77.

Post DM, Layman CA, Arrington DA, Takimoto G, Quattrochi J, Montaña CG (2007) Getting to the fat of the matter: Models, methods and assumptions for dealing with lipids in stable isotope analyses. Oecologia 152:179–189.

Reisinger RR, Landman M, Mgibantaka N, Smale MJ, Bester MN, De Bruyn PJN, Pistorius PA (2018) Overlap and temporal variation in the diets of sympatric Antarctic and Subantarctic fur seals (*Arctocephalus* spp.) at Marion Island, Prince Edward Islands. Polar Res 37:1451142.

Schoombie S, Crawford RJM, Makhado AB, Dyer BM, Ryan PG (2016) Recent population trends of sooty and light-mantled albatrosses breeding on Marion Island. Afr J Mar Sci 38:119 – 127.

Tickell WLN, Pinder R (1975) Breeding biology of the black-browed albatross *Diomedea melanophris* and grey-headed albatross *D. chrysostoma* at Bird Island, South Georgia. Ibis 117:433–451.
